# Identification and characterization of CircRNA-associated CeRNA networks in moso bamboo under nitrogen stress

**DOI:** 10.1186/s12870-023-04155-5

**Published:** 2023-03-14

**Authors:** Chenglei Zhu, Tingting Yuan, Kebin Yang, Yan Liu, Ying Li, Zhimin Gao

**Affiliations:** 1 Institute of Gene Science and Industrialization for Bamboo and Rattan Resources, International Centre for Bamboo and Rattan, Beijing, 100102 China; 2 Key Laboratory of National Forestry and Grassland Administration/Beijing for Bamboo &, Rattan Science and Technology, Beijing, 100102 China

**Keywords:** *Phyllostachys edulis*, Circular RNAs, Nitrogen stress, CeRNA networks

## Abstract

**Background:**

Nitrogen is a macronutrient element for plant growth and development. Circular RNAs (circRNAs) serve as pivotal regulators for the coordination between nutrient supply and plant demand. Moso bamboo (*Phyllostachys edulis*) is an excellent plant with fast growth, and the mechanism of the circRNA-target module in response to nitrogen remains unclear.

**Results:**

Deep small RNA sequencing results of moso bamboo seedlings under different concentrations of KNO_3_ (N0 = 0 mM, N6 = 6 mM, N18 = 18 mM) were used to identify circRNAs. A total of 549 circRNAs were obtained, of which 309 were generated from corresponding parental coding genes including 66 new ones. A total of 536 circRNA-parent genes were unevenly distributed in 24 scaffolds and were associated with root growth and development. Furthermore, 52 differentially expressed circRNAs (DECs) were obtained, including 24, 33 and 15 DECs from three comparisons of N0 vs. N6, N0 vs. N18 and N6 vs. N18, respectively. Based on integrative analyses of the identified DECs, differentially expressed mRNAs (DEGs), and miRNAs (DEMs), a competitive endogenous RNA (ceRNA) network was constructed, including five DECs, eight DEMs and 32 DEGs. A regulatory module of PeSca_6:12,316,320|12,372,905-novel_miR156-PH02Gene35622 was further verified by qPCR and dual-luciferase reporter assays.

**Conclusion:**

The results indicated that circRNAs could participate in multiple biological processes as miRNA sponges, including organ nitrogen compound biosynthesis and metabolic process regulation in moso bamboo. Our results provide valuable information for further study of circRNAs in moso bamboo under fluctuating nitrogen conditions.

**Supplementary Information:**

The online version contains supplementary material available at 10.1186/s12870-023-04155-5.

## Background

Circular RNAs (CircRNAs) are an important class of endogenous noncoding RNAs derived from mRNA precursor back-splicing and were reported more than four decades ago [1]. With the rapid development of high-throughput sequencing technology and the high efficiency of large data analyses, circRNAs have been widely identified in plants. There were 6,012, 12,037, 5,372 and 895 circRNAs identified in Arabidopsis (*Arabidopsis thaliana*), rice (*Oryza sativa*), soybean (*Glycine max*), and moso bamboo, respectively [2–4]. Comparison analysis of Arabidopsis and rice showed that parent genes of circRNAs with over 700 exons were orthologs, indicating the conservation of circRNAs in plants [2]. An increasing number of studies have reported that circRNAs are linked to important biological processes, including plant growth, development and flowering. Research has shown that circRNAs derived from fruit pigment biosynthesis genes regulate tomato (*Solanum lycopersicum*) fruit ripening [5]. Moreover, circRNAs were also shown to be involved in the regulation of wheat (*Triticum aestivum*) root length [6]. A total of 849 circRNAs were reported to participate in moso bamboo shoot fast growth [4]. In addition, studies have proposed that circRNAs are likely to be involved in biotic/abiotic stresses. A total of 280 circRNAs participating in the *Verticillium wilt* response in cotton (*Ossypium hirsutum*) were reported [7]. Large-scale profiles revealed that circRNAs responded to drought stress in maize (*Zea mays*), Arabidopsis and moso bamboo [8, 9]. These findings indicated that circRNAs might play crucial roles in the regulation of plant growth and development, and stress response.

As an important macronutrient, nitrogen is an essential component of nucleic acids, proteins, cofactors and phytohormones in plants. The function of nitrogen has been clearly exemplified by its effects on leaf expansion and flowering [10, 11]. It is also clear that nitrogen nutrition has an impact on root growth and development [12]. The processes including nitrogen uptake, transportation, assimilation and remobilization are complex, and many nitrogen-responsive genes have been identified [13]. It was reported that 10,422 genes were identified at the early stage of low nitrogen stress in rice [14]. Previous reports on nitrogen regulatory genes mainly focused on transcription factors (TFs) and miRNAs. During the last few years, an increasing number of TFs, such as MADS-box, LBD and NLP, have been reported to be central components in regulating the nitrate response in plants [15–17]. Some studies have reported changes in the expression levels of different miRNA families under nitrogen limitation conditions. Analysis of miRNA expression in maize seedlings showed that miR169, miR171, miR394, and miR398 were differentially expressed under N-deficiency stress [18]. Similarly, under nitrogen starvation, miR160 and miR167 were observed to mediate the growth and development of lateral roots in Arabidopsis [19, 20]. Furthermore, as important noncoding RNAs, circRNAs also play considerable roles in macronutrient stress. In maize, some circRNAs exhibited differential expression under high-nitrogen and low-nitrogen conditions, and a coexpression network of circRNA-miRNA-mRNA was constructed, suggesting that circRNAs might play a role in responding to nitrogen starvation stress [21]. Similar results were also reported in rice under phosphate starvation stress [22]. In poplars (*Populus* × *canescens*), circRNAs are involved in wood formation and chemical properties in acclimation to low nitrogen availability [23]. These findings showed that circRNAs could play significant roles in responding to nitrogen and regulating gene expression. However, research on the regulatory mechanism of circRNA is still in its infancy, and there have been relatively few studies verifying the regulatory network of competitive endogenous RNAs (ceRNAs).

Moso bamboo, one of the most representative bamboo species, with 4.68 million ha of forest area, accounts for 72.96% of the total bamboo forest area in China [24]. Moso bamboo has the characteristics of fast growth, strong regeneration ability and excellent mechanical strength, and has been a promising substitute for wood [25]. Noncoding RNAs were reported to participate in shoot growth, lignification, and drought stress responses [26, 27]. Nitrogen is an inorganic nutrient affecting the rapid growth of moso bamboo [28]. The enzyme activity and molecular mechanism of nitrogen metabolism have attracted much attention in moso bamboo [29, 30]. *AMT*s, *NPF*s, and *NLP*s are involved in nitrogen metabolism [31–33]. Moreover, it had been reported that circRNAs might participate in shoot growth of moso bamboo and drought stress [4, 9], and circRNA responses to nitrogen in plants had also been reported [34, 35]. However, most studies had focused on functional genes in bamboos [26]. The functions of circRNAs in bamboo roots under treatment with different nitrogen concentration are still unclear. In this study, high-throughput sequencing was used to identify and predict circRNAs in the roots of moso bamboo exposed to three concentration s of nitrogen [0 mM KNO_3_ (N0), 6 mM KNO_3_ (N6, control) and 18 mM KNO_3_ (N18)] [36]. We identified the differentially expressed circRNAs (DECs) and annotated the host protein-coding genes of these DECs. We also built a core regulatory module of circRNA-miRNA-mRNA, which was further validated by quantitative real-time PCR (qPCR) and luciferase reporter assays. Our study provides new insights into the ceRNA regulatory mechanism of moso bamboo in response to nitrogen stress.

## Results

### Characterization of circRNAs in moso bamboo under nitrogen stress

**Fig. 1:**
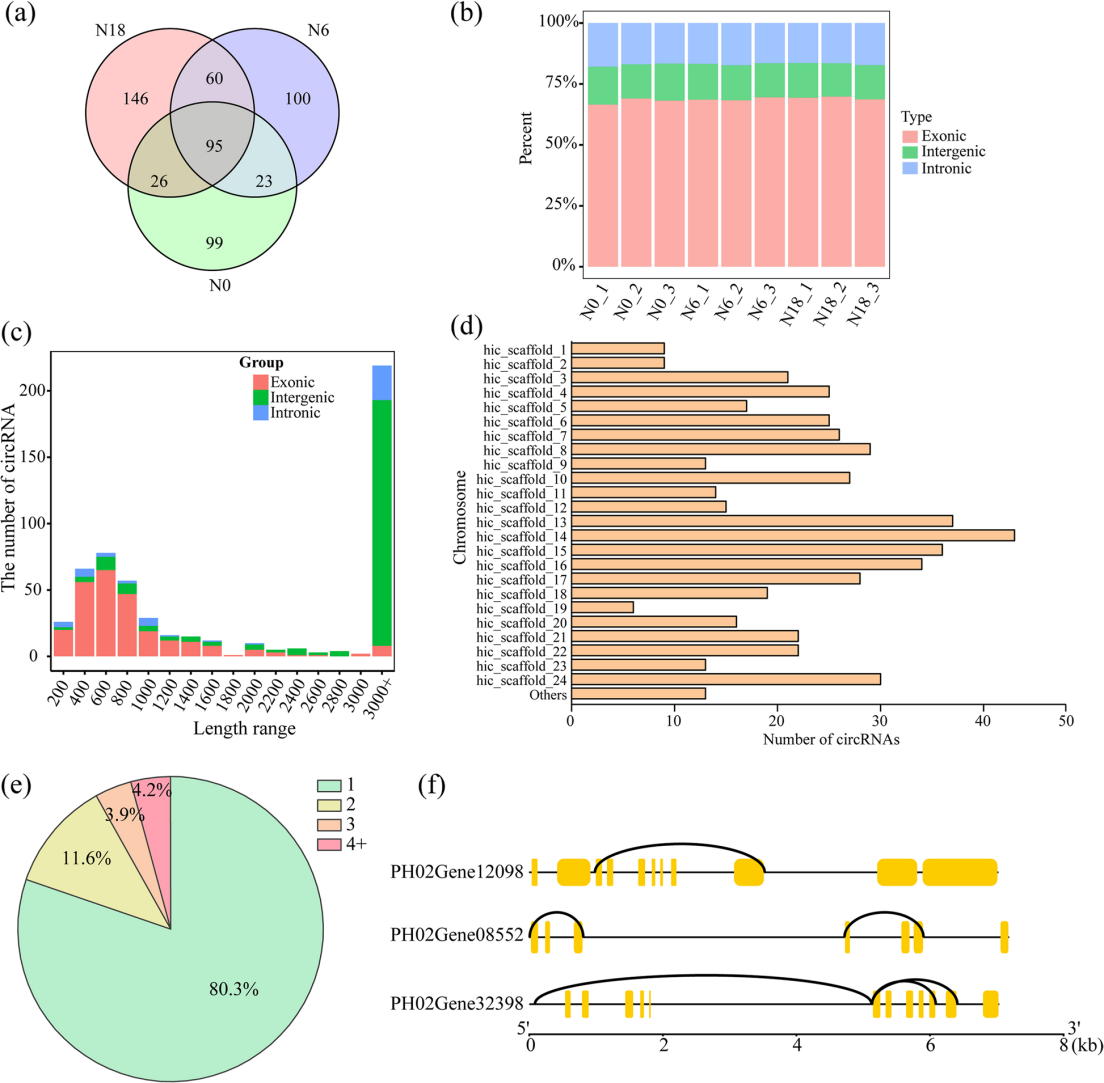
Global analysis of circRNAs in moso bamboo under nitrogen treatment. **a** Venn diagram showing the number and distribution of circRNAs detected in three comparisons. **b** Accumulative bar diagram showing the type of circRNAs in each sample. **c** Length range of moso bamboo circRNAs in three groups. **d** Histogram showing the number of circRNAs detected in each chromosome. **e** Pie chart showing percentage of circRNAs produced from the same host-gene. 1, 2, 3, and 4 + indicated that the numbers of circRNAs produced by one host-gene. **f** Examples of three host-genes that produced different members of circRNAs

To identify N-responsive circRNAs in moso bamboo, nine sets of sequencing data were collected from our previous study. A total of 482,510,256 clean reads were generated from nine samples. After removing adapter and low-quality sequences, as well as primer sequences, raw reads were mapped to the moso bamboo genome [37]. The guanine and cytosine (GC) contents were approximately 53.72%, and Q30 was greater than 93.99% (Table S1). A total of 549 circRNAs were detected from samples using CIRI tools (Fig. 1a). In addition, 95 circRNAs were found in all three comparisons. According to the genomic loci, most moso bamboo circRNAs were composed of exons (Fig. 1b). The length of circRNAs varied across a wide range; the main exonic circRNA lengths were between 200 bp and 1,000 bp, and the main intergenic circRNAs were > 3,000 bp (Fig. 1c), indicating that there might be more than one miRNA binding site and multiple RNA-binding proteins per circRNA. The number of circRNAs on each chromosome was counted (Fig. 1d). For example, the abundance and distribution of circRNAs on chromosomes 13, 14 and 15 were much greater than those on other chromosomes. Additionally, 309 circRNAs were derived from 272 host genes, of which 80.26% produced only one circRNA, and the rest produced more than one circRNA, with the largest number being nine circRNAs produced by one host gene (Fig. 1e). The same host gene generated various circRNAs through alternative splicing, and these alternative circRNAs were related to each other in one of three ways: no association, or one contained the other, or one had a similar splice site and the other had a different splice site (Fig. 1f).

### Confirmation of circRNAs in moso bamboo

**Fig. 2:**
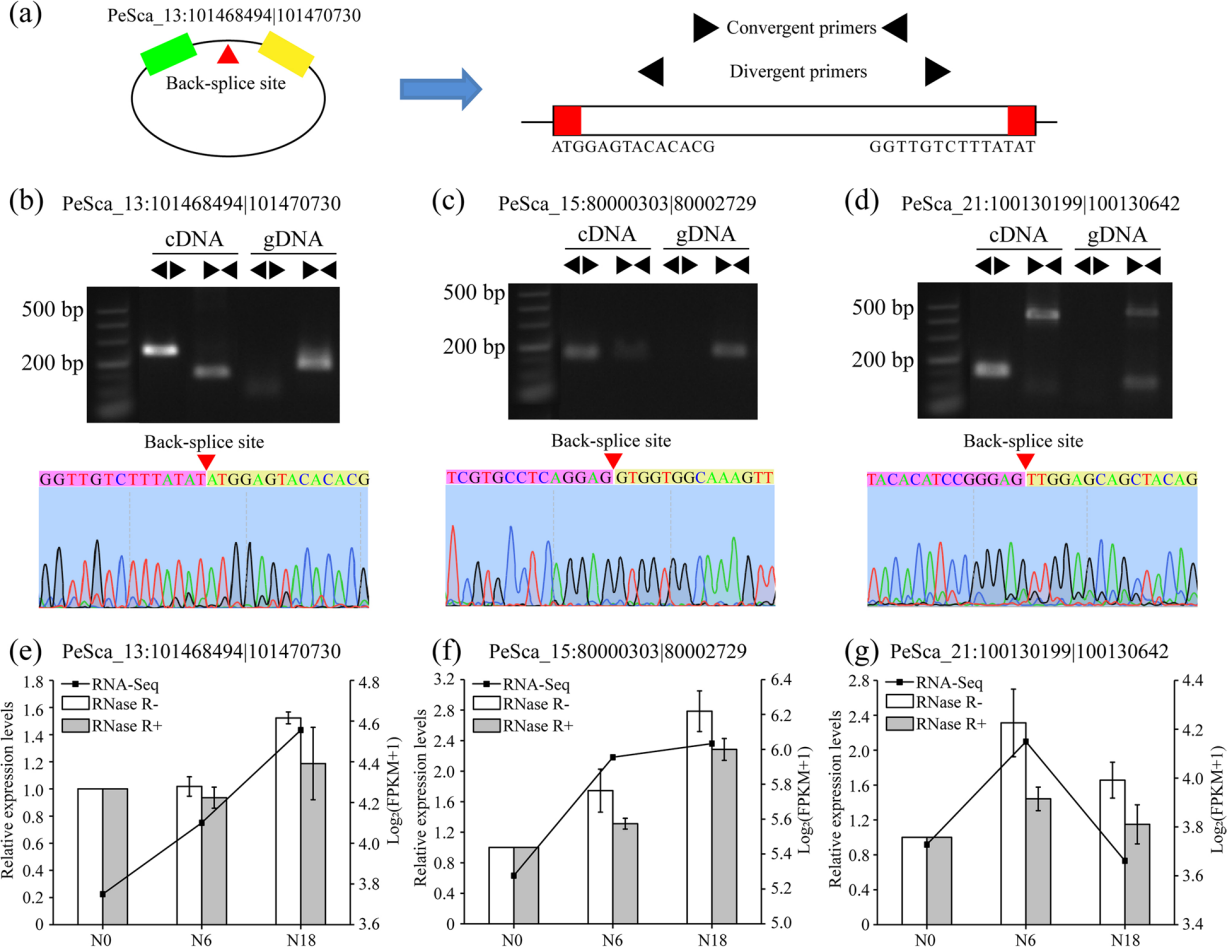
Validation of circRNAs. **a** The circRNA (PeSca_13:101,468,494|101,470,730) exemplified the validation strategy. According to the sequence of PeSca_13:101,468,494|101,470,730, two sets of arrows indicate two sets of amplification primers, which were designed to confirm head-to-tail back-splicing by PCR and Sanger sequencing. **b**-**d** Validation of PeSca_13:101,468,494|101,470,730, PeSca_15:80,000,303|80,002,729, and PeSca_21:100,130,199|100,130,642 by PCR and Sanger sequencing. Convergent primers amplified the circRNAs in both cDNA and gDNA. Divergent primers successfully amplified the circRNAs in cDNA but not those in gDNA. **e**–**g** Relative expression of three selected genes validated by qPCR. Error bars indicated standard deviation (SD) of three technical repeats. RNase R + and RNase R- represented total RNA treated with and without RNase R, respectively

To confirm the authenticity of circRNAs existing in moso bamboo, three highly expressed circRNAs were selected randomly for experimental validation by using convergent and divergent primers (Table S2) for polymerase chain reaction (PCR) and Sanger sequencing. Because the structure of circRNAs is different from that of linear RNAs, first-strand complementary DNA (cDNA) and genomic DNA (gDNA) samples were used for amplification (Fig. 2). Convergent primers from three circRNAs produced a single distinct band in both cDNA and gDNA samples (Fig. 2a), and PCR products of divergent primers of three circRNAs were detected only in cDNA samples, suggesting the presence of back-splicing junctions but not genomic rearrangements. In addition, PCR products of divergent primers were further detected by Sanger sequencing to confirm the back-splicing junctions (Fig. 2b-d). In addition, the qPCR results of these genes showed that the expression patterns were consistent with the RNA-seq results (Fig. 2e-g).

### Analysis of differentially expressed circRNAs (DECs) in moso bamboo under nitrogen stress

**Fig. 3:**
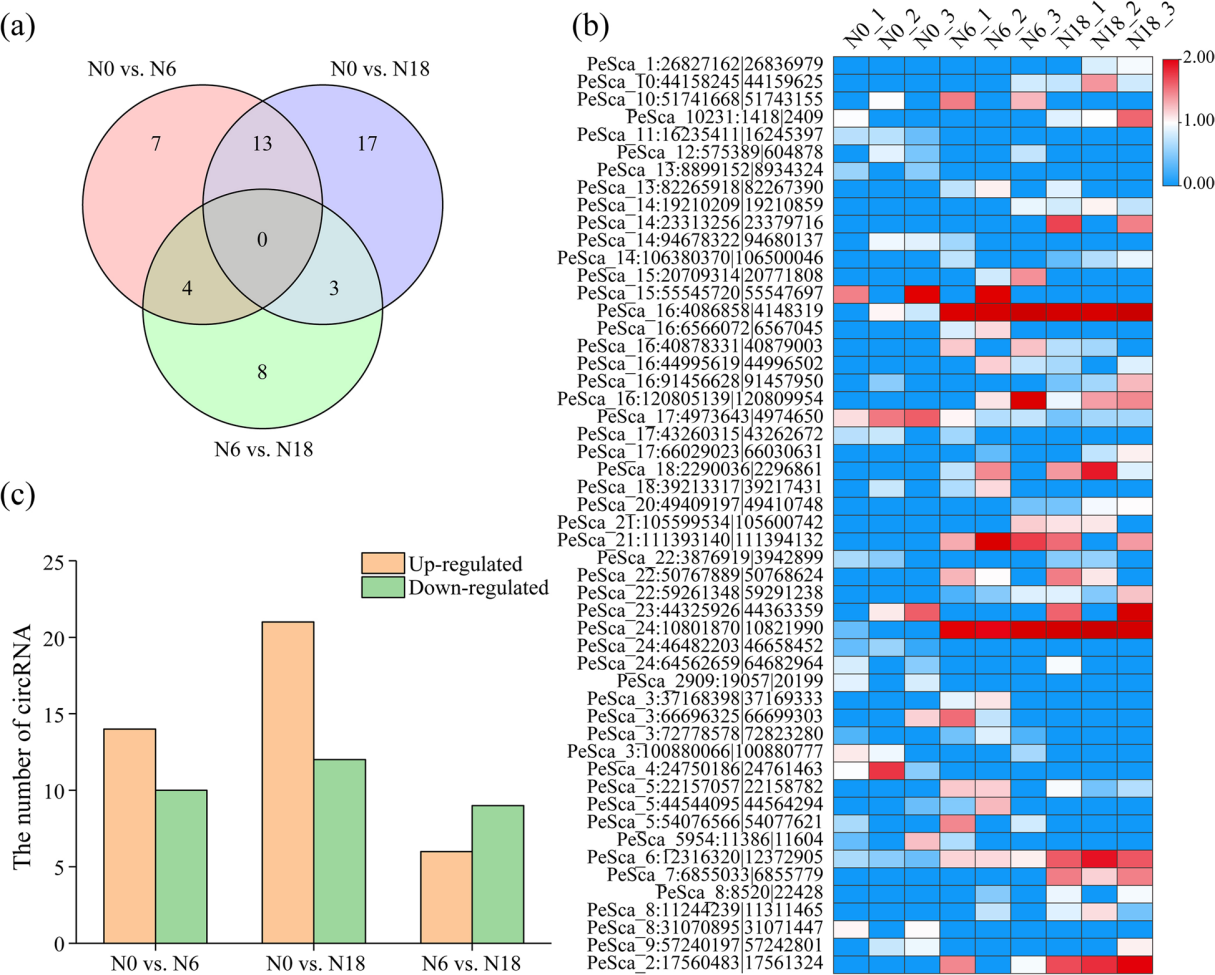
Differentially expressed analysis of circRNAs involved in nitrogen stress. **a** Venn diagram of circRNA distribution in different comparisons respectively. **b** Heatmap of expression of all DECs in nine samples. **c** Specific expression distribution of DECs in three comparisons

The expression specificity of circRNAs provides insights into their biological function. To reveal the expression pattern of circRNAs in moso bamboo under different nitrogen concentrations, the 549 circRNAs identified in three comparisons were filtered by retaining only those detected in at least two biological replicates. The filtered circRNAs were used for the following differential expression analysis. Based on the screening criteria, 24, 33, and 15 DECs were identified in three comparisons (N0 vs. N6; N0 vs. N18 and N6 vs. N18), respectively. Eventually, a total of 52 DECs were obtained (Fig. 3a), and the expression of DECs in each group was also displayed (Fig. 3b). Fourteen DECs were upregulated while 10 DECs were downregulated in N0 vs. N6, and 21 DECs were upregulated while 12 DECs were downregulated in N0 vs. N18 (Fig. 3c and Table S3). Obviously, the number of DECs in N6 vs. N18 was the lowest, which might imply little difference in circRNAs between the N6 and N18 treatments. The DECs identified above might have specific functions in moso bamboo under nitrogen stress.

### Functional prediction of host-genes for circRNAs in moso bamboo

A majority of circRNAs have unclear molecular functions, although some circRNAs have been shown to be involved in the regulation of their host-gene expression. According to circRNA alignment to genomic location, the host-genes of 52 DECs were obtained. A total of 20 host-genes were predicted and annotated (Table S4), whereas the remaining 32 host genes did not have parental coding genes. To further investigate the potential functions of DECs involved in nitrogen uptake and utilization of moso bamboo, functional enrichment analysis of these 20 predicted host genes was performed.

**Fig. 4:**
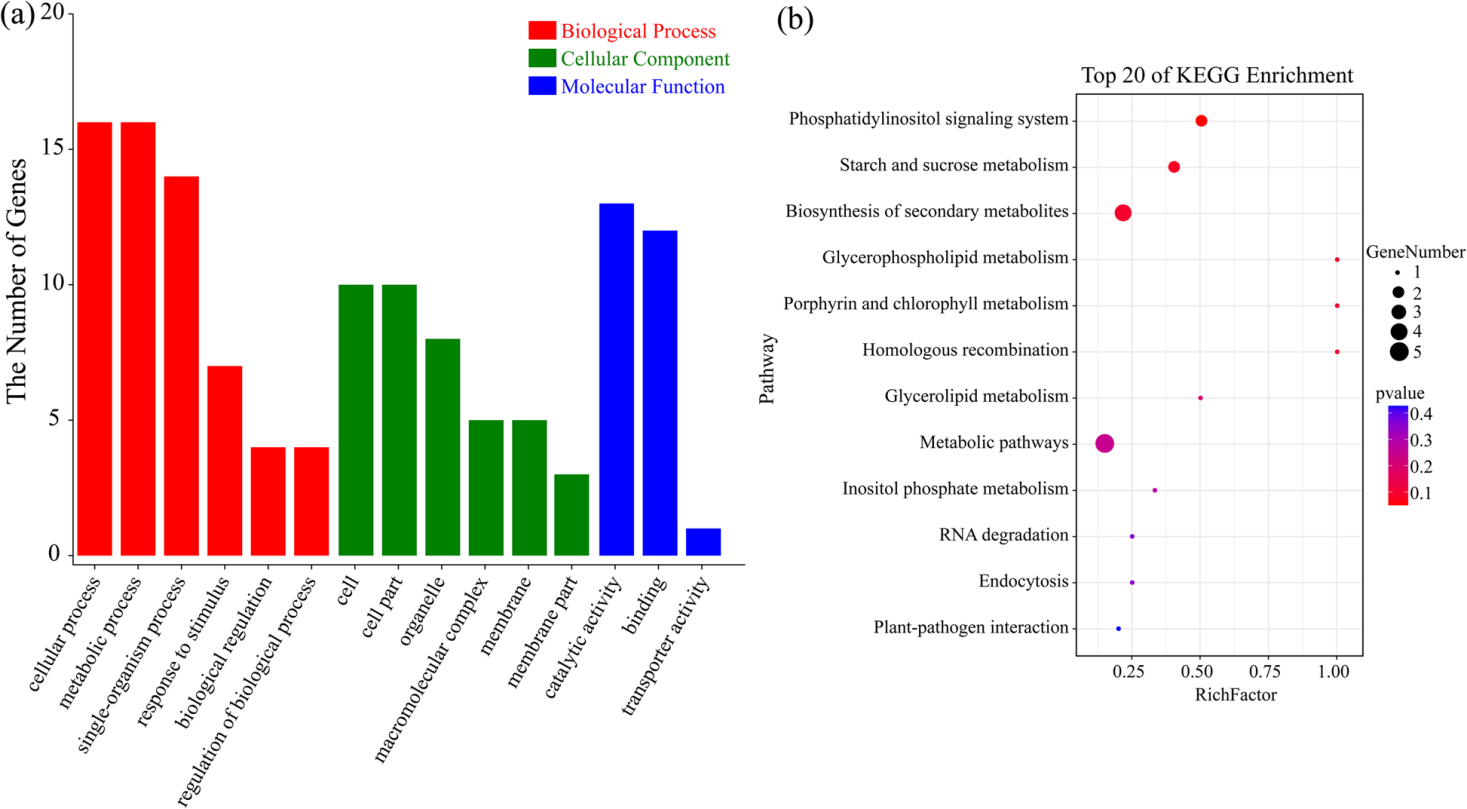
GO and KEGG enrichment analyses of host-genes for DECs. **a** The most enriched GO terms of the host-genes. **b** The most enriched KEGG pathways of the host-genes

Gene Ontology (GO) enrichment analysis showed that these host-genes were assigned to 29 GO terms, among which 16, 10 and three GO terms were classified into biological process, cellular component, and molecular function, respectively (Fig. 4a). Under biological process, ‘cellular process (GO:0,008,152)’ and ‘metabolic process (GO:0,009,987)’ were the most representative GO terms, followed by the category of ‘single-organism process (GO:0,044,699)’. Most importantly, many cellular components were found to be closely related to ‘cell (GO:0,005,623)’, ‘cell part (GO:0,044,464)’, and ‘organelle (GO:0,043,226)’. In the molecular function group, the two main typical categories were ‘binding (GO:0,005,488)’ and ‘catalytic activity (GO:0,003,824)’. Kyoto Encyclopedia of Genes and Genomes (KEGG) pathway analysis was also performed to further explore the functions of the host-genes of DECs. The results revealed that host-genes of circRNAs were significantly enriched in three pathways, including ‘phosphatidylinositol signaling system (ko04070)’, ‘starch and sucrose metabolism (ko00500)’, and ‘biosynthesis of secondary metabolites (ko01110)’ (Fig. 4b). This result implied that host genes of circRNAs in moso bamboo might be involved in material synthesis and energy metabolism processes as well as signal transduction.

### Putative functions of circRNAs acting as miRNA sponges in moso bamboo

**Fig. 5:**
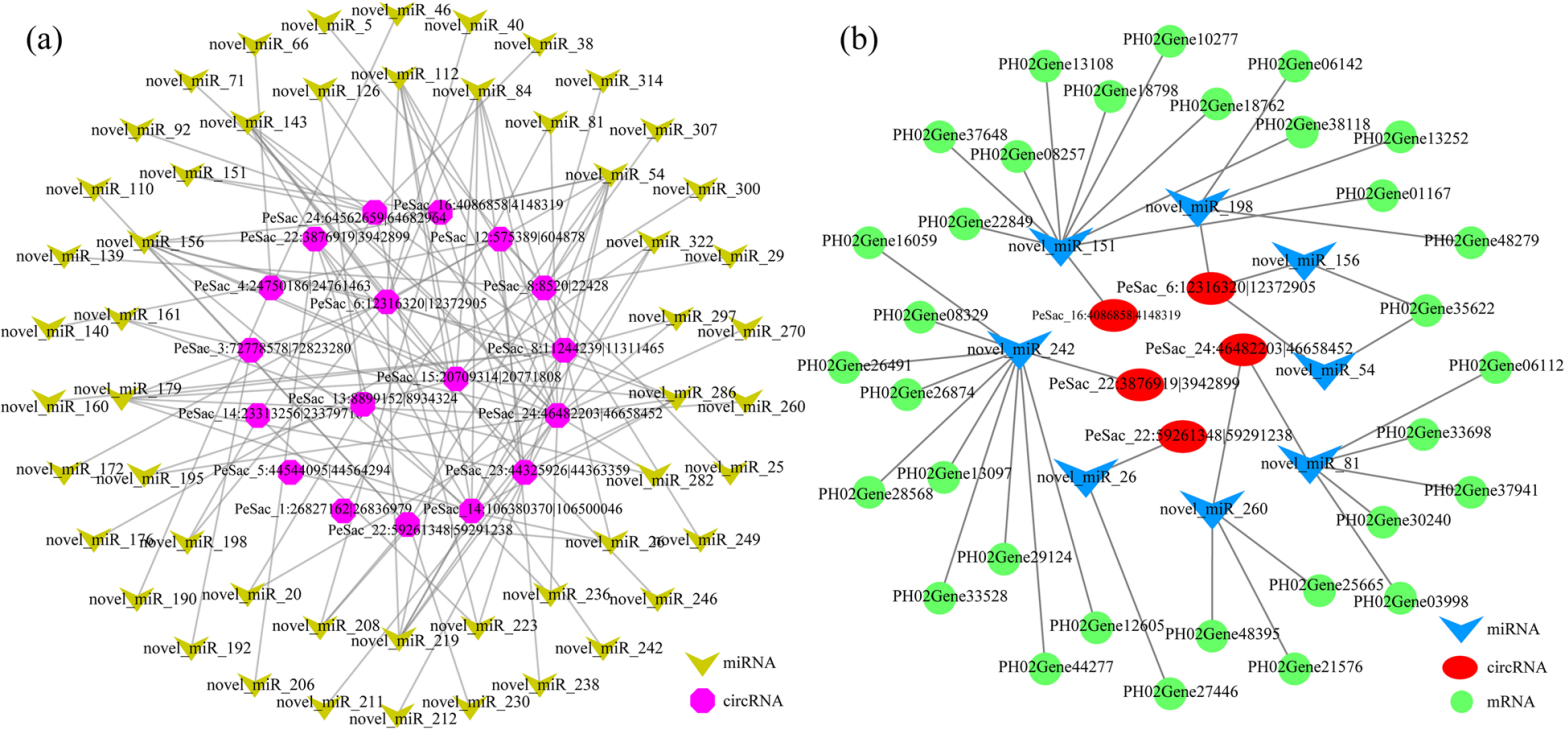
CeRNA interaction networks in moso bamboo. **a** The circRNA-miRNA networks comprising 18 DECs (purple circle) and 53 DEMs (yellow V-shape) in moso bamboo. **b** The ceRNA networks formed by eight DECs (red ellipse), five DEMs (blue V-shape) and their co-expressed 32 differentially expressed genes (DEGs) (green circle) in moso bamboo under nitrogen stress

A large number of studies have shown that circRNAs can act as sponges to competitively bind to miRNAs, thereby inhibiting miRNA binding to mRNAs and regulating gene expression. To detect the function of circRNAs as miRNA sponges, the TargetFinder software was used to predict potential miRNA binding sites and identify bamboo miRNAs targeting circRNAs. The N-responsive candidate miRNAs were analyzed to screen the miRNA-circRNA pairs. We found that 22 DECs contained 118 miRNA binding sites, and a total of 373 miRNA-circRNA interactions were identified (Fig. S1 and Table S5). Thus 22 DECs might function as miRNA sponges in response to nitrogen stress in moso bamboo. Among these 118 miRNAs, we found that some miRNAs, such as novel_miR_64, novel_miR_112, and novel_miR_156, could bind several circRNAs. Moreover, we also found that some miRNAs, such as ata-miR156b-3p, novel_miR_92, and novel_miR_260, corresponded to only one circRNA. Further analyses showed that 53 differentially expressed miRNAs (DEMs) were obtained from 118 miRNAs, and 114 DEM-DEC interactions were identified (Fig. 5a). Among these DEMs, 29 were classified into 14 miRNA families, such as miR160, miR169, miR398 and miR528.

It has been reported that circRNAs can act as sponges of miRNAs to regulate the target mRNAs of corresponding miRNAs by ceRNA networks. In addition, circRNAs were reported to have expression patterns similar to those mRNAs in ceRNA networks. On the basis of the ceRNA hypothesis, we searched for the same miRNA binding sites in the circRNA-gene pairs. CircRNA-miRNA-mRNA networks were constructed to reveal the functions of circRNAs acting as miRNA sponges. As shown in Fig. 5b and Table S6, a total of five DECs were identified as potential miRNA sponges that regulated the expression of target genes through ceRNA networks. It was found that three circRNAs might sponge three miRNAs to regulate the expression of mRNAs. An upregulated DEC, PeSca_6:12,316,320|12,372,905 may bind with three miRNAs to regulate the expression of PH02Gene35622, PH02Gene06142 and PH02Gene48279.

The functional annotation of 32 protein-coding genes of mRNA from ceRNA networks was performed. The KOG and COG annotation suggested that four mRNAs might be associated with amino acid transport and metabolism, such as serine carboxypeptidase and aromatic amino acid decarboxylase. Four target genes were annotated to be related to carbohydrate transport and metabolism (Table S7). Moreover, TFs and protein kinases were also annotated by the Pram database. Thus, circRNAs associated with the nitrogen response might play important roles in nitrogen assimilation by using a ceRNA regulatory network to regulate some mRNAs related to carbon and nitrogen metabolism.

### Validation of ceRNA regulatory modules by qPCR and dual-luciferase reporter assay

**Fig. 6:**
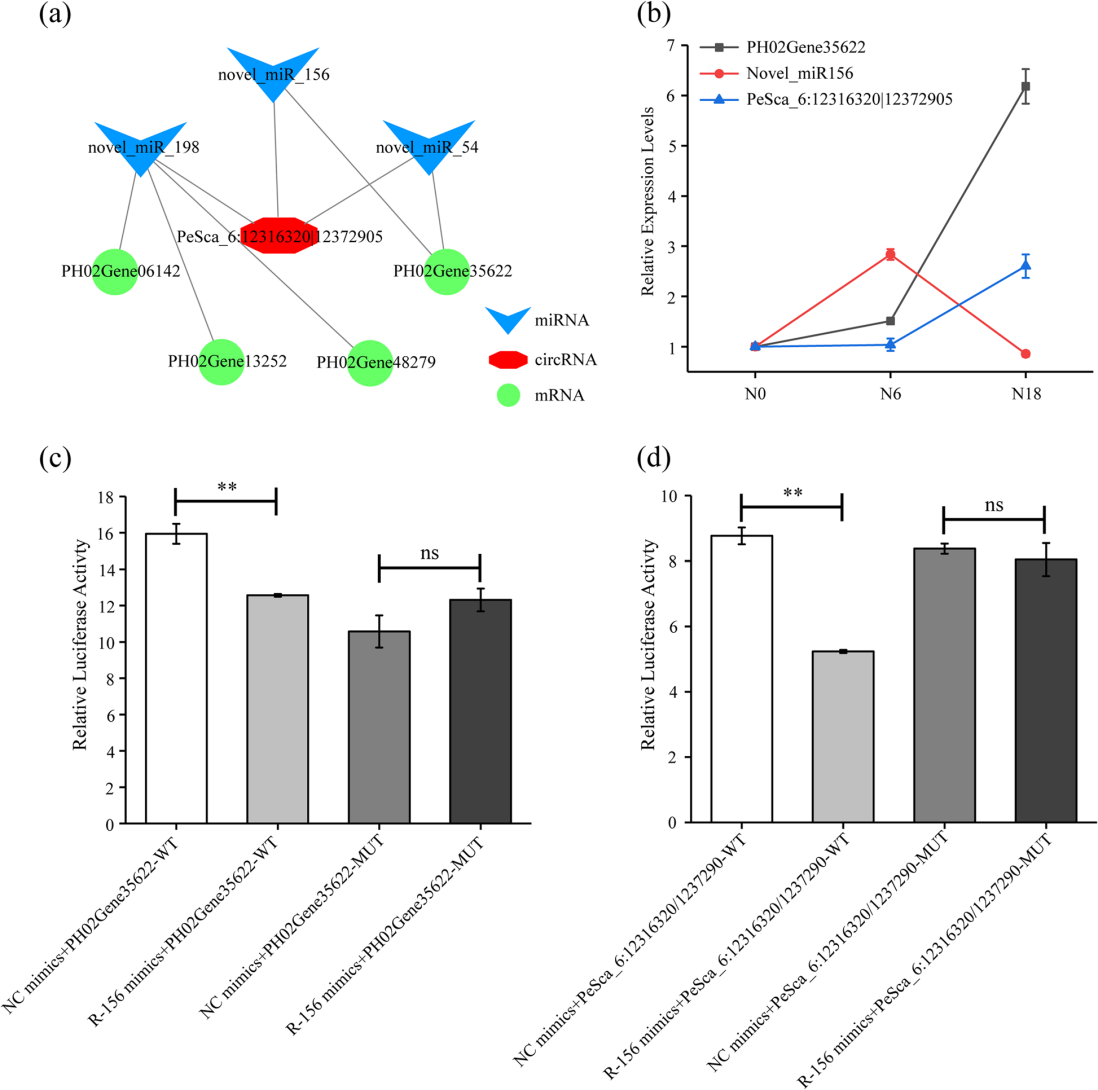
A regulatory module of circRNA-miRNA-mRNA was verified by qPCR and luciferase reporter assay. **a** The model of circRNAs regulating mRNAs through ceRNA module to participate in nitrogen absorption of moso bamboo. **b** Expression analysis of PeSca_6:12,316,320|12,372,905, novel_miR156 and PH02Gene35622 in moso bamboo under nitrogen stress by qPCR. **c**-**d** Verification of the correlation between PeSca_6:12,316,320|12,372,905, novel_miR156 and PH02Gene35622 using a dual-luciferase reporter assay. NC and R-156 minmics indicated negative control and recombinant plasmid of novel_miR156, respectively. WT indicated plasmid containing wild-type sequences of mRNAs or circRNAs, and MUT indicated plasmid containing mutant type sequences of mRNAs or circRNAs, respectively. Error bars indicated SD of three technical repeats. Statistical analyses were performed by* t*-test, **: *p* < 0.01, ns: *p* > 0.05

Based on the ceRNA modules and expression levels of circRNAs, miRNAs and mRNAs (Fig. 6a), we validated a ceRNA regulatory module containing a highly expressed circRNA (PeSca_6:12,316,320|12,372,905) and a co-expressed mRNA (PH02Gene35622: serine carboxypeptidase) as well as a coupled miRNA (novel_miR156) by qPCR and dual-luciferase reporter assays. The qPCR results showed that novel_miR156 ws upregulated under N6 compared with under N0, but it was downregulated under N18 compared with under N0 and N6. Its target circRNA (PeSca_6:12,316,320|12,372,905) and gene (PH02Gene35622) were upregulated under N6 and N18 compared with under N0 (Fig. 6b). The qPCR of ceRNA pairs further validated the results of the high-throughput sequencing. Additionally, the dual-luciferase reporter assay indicated that novel_miR156 specifically bound to PeSca_6:12,316,320|12,372,905 and PH02Gene35622. The luciferase activity of the novel_miR156 mimics + mRNA/circRNA-WT group was lower than that of the NC mimics + mRNA/circRNA-WT group (*p* < 0.01) in the transfected cells; however, there was no significant difference between the two mutant groups (Fig. 6c-d). These results provide evidence for elucidating the relationship between the genetic elements in the circRNA-miRNA-mRNA regulatory module.

## Discussion

As common noncoding RNAs, circRNAs have been discovered in various animals and plants. In higher plants, an increasing number of circRNAs have been identified from Arabidopsis, rice, soybean, and moso bamboo [2–4]. Previous studies have not only demonstrated that circRNAs play important roles in many biological and developmental processes but have also attested that circRNAs are crucial in responding to various biological and abiotic stresses [6, 38–40]. As a key essential macronutrient, nitrogen plays important functions in the growth and development of plants, and its availability has a strong influence on biological processes [41]. Previous studies showed that circRNAs participated in nitrogen absorption in maize seedlings [21]. Therefore, revealing the characteristics of circRNAs under different nitrogen treatments is crucial to further understand the function of noncoding RNAs in plants. In this study, 549 circRNAs were identified and found to be widely distributed in the moso bamboo genome (Fig. 1a). It is worth noting that 80.3% of circRNAs were produced by only one host gene (Fig. 1e), which was generally consistent with studies in tea (*Camellia sinensis*) and sea buckthorn (*Hippophae rhamnoides*) [42, 43]. Furthermore, some host genes producing more than one circRNA were found in this study. Similar results also appeared in maize seedlings under deficient nitrogen, and these circRNAs were produced by lariat derivation or by back-splicing in a single gene [21].

CircRNAs were divided into three types based on their location on the genome, including exonic, intronic and intergenic circRNAs [44]. Here, exonic circRNAs were predominant in moso bamboo (Fig. 1b), which was in accordance with previous studies in tomato, maize and Arabidopsis [2, 21, 45]. In contrast, 51% of total circRNAs were intergenic circRNAs in kiwifruit (*Actinidia chinensis*) plants [39]. When it came to wheat, things were different. More than 70% of total circRNAs were exonic circRNAs in roots [6]. However, more intergenic circRNAs were identified in the leaves of wheat under dehydration stress [38]. Similar results were found in soybean [3]. The results might be attributed to following factors: (i) The sequencing depth might influence the numbers of identified circRNAs. Approximately 88 circRNAs were isolated from only 90 M read sequencing data of wheat seedling leaves [38], while more than 1,000 circRNAs were identified based on 300 M paired-end reads of wheat roots [6]. (ii) The genome size and available gene numbers should be the main reason for the different circRNA percentages. The number of moso bamboo genes is 51,074, and the genome size is 1,910 Mb [37]. Comparatively, there were 27,655 and 35,679 genes with 389 Mb and 120 Mb genome sizes for rice and Arabidopsis, respectively. (iii) Exonic and intronic circRNAs with different proportions might be due to whole genome duplication events and multiple copy numbers of genes, such as soybean and moso bamboo [46, 47]. (iv) CircRNAs showed significant tissue-/stress- specific expression patterns in plants. The material used in the present study was seedling roots of moso bamboo exposed to nitrogen stress. The distribution of diverse circRNA types might be different in various tissues of moso bamboo.

Nitrogen is the most important essential macronutrient and plays a preliminary role in plant germination, growth, development and propagation [41]. Nitrogen transport, utilization and assimilation have been extensively studied in moso bamboo [30, 48]. However, the roles of circRNAs in the nitrogen response process of moso bamboo have not been reported. The results reported that nitrogen treatment in wheat altered the expression profiles of circRNAs [21]. In this study, 52 circRNAs were identified as DECs between nitrogen-treated seedlings (Fig. 3a). Interestingly, we found that some circRNAs, such as PeSca_16:4,086,858|4,148,319, PeSca_16:12,085,139|120,809,954, and PeSca_24:10,801,870|10,821,990, were specifically expressed in the N6 and N18 groups, while PeSca_4:24,750,186|24,761,463 was specifically expressed in the N0 group (Fig. 3b). The fluctuation of circRNA abundance after nitrogen treatment in moso bamboo might be related to the possible roles of circRNAs in response to nitrogen. Moreover, the results indicated that the expression of circRNAs was correlated with that of their host genes [2, 39]. It has been reported that overexpression of a circRNA can reduce the expression level of its parental gene [49]. A circRNA from *SEPALLATA3* was shown to regulate host-gene expression by R-loop formation [50]. In the present study, the results of GO and KEGG enrichment analyses confirmed that host genes of DECs were involved in various and important biological processes, including cellular and metabolic processes (Fig. 4). Similar results were found in wheat seedling roots under nitrogen treatment [21]. Thus, these results indicated that circRNAs in moso bamboo might have crucial functions in the synthesis of metabolites under different nitrogen conditions.

Many studies have reported that miRNAs participate in plant nitrogen metabolism. For example, 20 miRNAs were identified in peanut (*Arachis hypogaea*) in response to low nitrogen stress [9]. Some miRNAs were predicted to target certain circRNAs in maize under deficient nitrogen [21]. Some studies demonstrated that miR482, miR1512 and miR1515 were related to nitrogen fixation [51], and further research speculated that these miRNAs might target to circRNAs in soybean [3]. In the present study, a total of 18 DECs could act as 53 DEM sponges, and 114 miRNA-circRNA interactions were found at the same time (Fig. 5a), suggesting that these circRNAs might function as miRNA sponges to regulate gene expression in moso bamboo under nitrogen stress. Studies have shown that members of the miR166, miR169 and miR394 families might take part in nitrogen stress [18, 52, 53]. Similarly, novel_miR192, novel_miR26, novel_miR260 and novel_miR40, were also identified in this study. Thus, circRNAs might affect the expression of mRNAs by sponging miRNAs.

CeRNA networks play important roles in regulating gene expression. CircRNAs have been reported to act as miRNA sponges by sponging miRNAs to regulate the expression of their target genes [54]. In Arabidopsis, ceRNA networks participate in leaf development by regulating the expression of genes [55]. Further analysis of miRNA target genes associated with nitrogen responsiveness revealed a regulatory network containing 40 interaction pairs between five circRNAs, eight miRNAs and 32 mRNAs in moso bamboo roots based on the ceRNA theory (Fig. 5b). Functional analysis of target genes indicated that these circRNAs could participate in amino acid metabolism and posttranslational modification. TFs were also identified in this study. Similar results were found in the ceRNA network of wheat [21]. The insufficiencies of this study were that no circRNA related genes associated with nitrogen uptake, transport and assimilation were identified. There were two speculative reasons: (i) The characterization of low-expression levels of circRNAs. The expression of circRNA was one hundredth of that of its host-gene [56], so more circRNAs were difficult to detect in ordinary sequencing. (ii) The sequencing RNAs were derived from seedling roots in this study. CircRNAs associated with nitrogen assimilation and utilization might occur in other organs, such as shoots and leaves [23, 42]. Therefore, further studies are still needed. The function of circRNAs has been elucidated over the last few years. CircRNAs might function as miRNA sponges to restrain translation of mRNA, and influence gene expression by regulating alternative splicing, or by interacting with RNA-binding proteins [57]. Based on the expression levels and the miRNA binding sites of circRNA and mRNA, a module of PeSca_6:12,316,320|12,372,905-novel_miR156-PH02Gene35622 was verified by qPCR and luciferase reporter assays (Fig. 6).

Based on above results, 18 DECs had miRNA binding sites, in which five DECs could form a ceRNA regulatory network with eight miRNAs and 32 target genes. We speculate that circRNAs play significant roles in amino acid metabolism of moso bamboo. These results provide new ideas for studying circRNAs in response to nitrogen in roots to regulate the plant growth and development of moso bamboo. However, the function of these circRNAs remains tentative, and the deeper functionality of circRNAs in moso bamboo needs further experimental validation. Furthermore, the relationship between circRNAs and their targeted miRNAs also needs further investigation in the process of nitrogen absorption in moso bamboo roots.

## Conclusion

In this study, a circRNA pool was constructed in moso bamboo under nitrogen stress by small RNA data, and 549 unique circRNAs were identified. Fifty-two DECs were obtained by the comparing moso bamboo roots under different nitrogen conditions, and the expression patterns of these DECs were investigated. The possible functions of these DECs were predicted according to their host-genes using GO and KEGG analyses, suggesting that these circRNAs might be involved in energy metabolism processes and signal transduction in moso bamboo. A total of 114 miRNA-circRNA interaction pairs were found in 18 DECs and 53 DEMs. A regulatory network of circRNA-miRNA-mRNA was constructed, containing five DECs, eight DEMs and 32 DEGs. Furthermore, a module of PeSca_6:12,316,320|12,372,905-novel_miR156-PH02Gene35622 was verified by qPCR and luciferase reporter assays. These results indicated that circRNAs might participate in the regulation of nitrogen response in moso bamboo. However, the function and regulatory effect of these circRNAs in moso bamboo requires further experimental verification.

## Methods

### Plant materials and dataset

The seedlings of moso bamboo grew in a favorable environment (photoperiod: 16-h light/8-h dark, photon density: ~ 300 µmol·m^−2^·s^−1^, temperature: ~ 28 °C and humidity: ~ 60%) for two months. Ninety well-grown seedlings with similar heights (∼15 cm) were selected, and the roots were cleaned and then placed into modified Kimura B solution with different concentrations of KNO_3_ (N0 = 0 mM, N6 = 6 mM, N18 = 18 mM) for two weeks [58]. The seedling roots were harvested and stored at -80 °C after quickly frozen [36]. Each nitrogen treated group contained three biological replicates. Total RNA was extracted from nine samples using the Kit (Tianmo, TR205-50, China) for transcriptome and small RNA sequencing. Library construction, quality control, and data processing were conducted as in previous studies [26, 27]. All small RNA and RNA-seq data sequences have been deposited in the Sequence Read Archive (SRA) under project IDs PRJNA797724 (https://www.ncbi.nlm.nih.gov/bioproject/PRJNA797724) (SRR17650178 ~ SRR17650186) and PRJNA797734 (https://www.ncbi.nlm.nih.gov/bioproject/PRJNA797734) (SRR17635191 ~ SRR17635199) [36]. The detailed information of SRA project is listed in Table S8.

### Bioinformatics identification of circRNAs in moso bamboo

Prior to genome-wide identification of circRNA in moso bamboo, an in-house Perl script was used to process all sequencing rate data in fastq format. All clean reads were obtained through filtering reads [59]. Then, the GC-content, Q20, and Q30 were calculated. All the resultant clean reads were mapped to the moso bamboo genome and annotation files relying on TopHat (v.2.0.9) software [60, 61]. CircRNAs were identified by CircRNA Identifier (CIRI, v.2.1.1) and find_circ tools (v.1.2) [62, 63]. Based on their genomic origins, the identified circRNAs were classified into three types: intergenic, intronic, and exonic circRNAs [44].

### Differentially expressed circRNA analysis and functional prediction of host-genes

To compare the expression of circRNAs in moso bamboo roots across three nitrogen treatments, circRNA expression levels were evaluated by the circular-to-linear ratio and junction read counts (RPM). StringTie (v.1.3.1) software was used to calculate the expression of protein-coding transcripts [64]. The circRNA expression levels were categorized into five levels (0–0.5, 0.5–1, 1–5, 5–10, and > 10), and the expression levels of host genes corresponded to five levels: 0–50, 50–100, 100–500, 500–2000, and > 2000 [56]. Differentially expressed circRNAs (DECs) between any two groups (N0 vs. N6; N0 vs. N18; N6 vs. N18) were analyzed using the DESeq R package (v.1.10.1) with Fold Change > 1.5 and *P* value < 0.05 according to Benjamini and Hochberg’s methods [65].

Since the function of circRNAs depends on their host genes, the correlation between one circRNA and its host gene is critical to study the function of circRNA. The GOseq R (v.1.46.0) package was used to identify the functions of host-genes of DECs, and GO terms were considered to be significantly enriched with a *P* value < 0.05 by DEGs [66]. KOBAS (v.3.0) software was used to conduct the statistical enrichment of DEC host-genes in KEGG pathways [66].

### Prediction of miRNA binding sites in circRNAs and co-expression analyses of circRNAs and mRNAs

There are many miRNA binding sites in the sequence of circRNAs. When they combine with miRNAs, the function of miRNAs can be limited. Eventually, circRNAs improved the inhibition of miRNA on target genes and increased the expression level of target genes. Therefore, to further study circRNA functions, we analyzed miRNA binding sites of circRNAs with TargetFinder (v.1.1.3) software [21, 67]. The regulatory network of miRNAs and circRNAs was visualized by using Cytoscape (v.3.7.1) software. Genes with the same expression pattern tend to have a similar function. Pearson correlation coefficients were used to construct co-expression network of circRNAs and mRNAs with an absolute value of correlation ≥ 0.9, and *P* < 0.01 [68].

### Construction of the circRNA-miRNA-mRNA regulatory network

The regulatory mechanism of ceRNAs has been a research hotspot in recent years, and ceRNAs can competitively bind the same miRNA to regulate gene expression levels through miRNA response elements (MRE). To further analyze the function of circRNAs in moso bamboo roots under nitrogen treatment, we first took the intersection of DEGs and DECs. Subsequently, the targeting relationships among the DECs, DEGs and mature miRNAs were analyzed to study the function of circRNA. Finally, based on ceRNA theory, we searched for the same miRNA binding site in circRNA-mRNA pairs, and a ceRNA regulatory network was constructed by circRNA-miRNA-mRNA pairs using Cytoscape software [21].

### Validation of circRNAs

Genomic DNA (gDNA) of bamboo roots was extracted using cetyltrimethylammonium bromide (CTAB). Total RNA from moso bamboo roots under three nitrogen treatments was extracted using the Total RNA Kit (Tianmo, TR205-50, China) according to the manufacturer’s protocol. Total RNA was treated with RNase R (2–3 units RNase R per μg RNA) at 37 °C for 15 min (Jisai, R0301, China). After treatment, two kinds of first-strand cDNA (RNase R + and RNase R-) were synthesized using a PrimeScript RT Reagent kit (Takara, RR037A, Japan) with the treated RNA and total RNA, respectively. Specific primers (divergent and convergent primers) were designed for the circRNAs by Primer Premier (v.5.0) software. PCR was performed with templates of gDNA and cDNA (RNase R +). The PCR products were further analyzed by Sanger sequencing (Ruibiotech, Beijing, China).

To validate the expression of circRNAs, qPCR was conducted with two types of cDNA (RNase R + and RNase R-) using the Roche Light Cycler 480 SYBR® Green I Master kit (Roche, 04,887,352,001, Germany) with specific primers (Table S2), and qPCR of the genes with cDNA (RNase R-) was conducted using the same reagents with specific primers (Table S2). *PeTIP41* was used as an internal control [69]. miRNA first-strand cDNA and qPCR of miRNAs were performed using the miRNA 1^st^ Strand cDNA Synthesis Kit (Aidlab, PC4801, China) and miRNA Universal SYBR® qPCR Master Mix (Aidlab, PC4901, China). *U6* snRNA was used as an internal control [70]. Three independent experiments were performed. The results were analyzed and visualized using Origin Pro 2017 and Adobe illustrator CC 2018 software.

Wild-type (WT) mature sequences of miRNAs, 200 bp flanking sequences of mRNAs and circRNAs, including the predicted splicing sites were synthesized artificially (Ruibiotech, Beijing, China), and mutant type (MUT) sequences of mRNAs and circRNAs after site-directed mutation of target sites (Non-synonymous nucleobase substitution) were synthesized artificially [27]. Then, all sequences were cloned into the pmirGLO vector (GeneCreate, Wuhan, China). After confirmation by sequencing, MUT and WT vectors were co-transfected with negative control (NC) mimics and miRNA mimics into 293 T cells, respectively [27]. After 48 h of transfection, the relative luciferase activity was measured with luciferase reporter assay kit (Beyotime, RG027, China), and then normalized to renilla luciferase activity. The proportion of firefly luciferase/renilla luciferase activity in each cell was used to quantify outcomes. Three independent experiments were performed. The NC, miRNA mimics, WT and MUT sequence information for the plasmids is listed in Table S9.

## Supplementary Information

**Additional file 1:** **Table S1.** Sequencing information of nine samples.

**Additional file 2:** **Table S2.** The primer sequences used in this study.

**Additional file 3**: **Table S3.** DECs analysis of three comparisons.

**Additional file 4:** **Table S4.** Functional annotation of 20 host-gene of DEGs.

**Additional file 5:** **Table S5.** 373 interactions of miRNA-circRNA.

**Additional file 6:** **Figure S1.** The circRNA-miRNA networks comprising the 22 DECs (blue square) and their targets of 118 miRNAs (red rhombus) in moso bamboo.

**Additional file 7**: **Table S6.** Information of DEC-DEM-DEG pairs.

**Additional file 8**: **Table S7.** Function annotation of 32 DEGs based on eight databases.

**Additional file 9**: **Table S8.** The detailed information of transcriptome and small RNA data.

**Additional file 10: Table S9.** The sequences of NC and miRNA mimics, as well as WT and MUT in plasmids.

## Data Availability

The datasets generated during the current study are available in the NCBI repository under the accession numbers PRJNA797724 (https://www.ncbi.nlm.nih.gov/bioproject/PRJNA797724) and PRJNA797734 (https://www.ncbi.nlm.nih.gov/bioproject/PRJNA797734).

## Abbreviations

AMTs: Ammonium transporters
cDNA: First-strand complementary DNA
ceRNA: Competitive endogenous RNA
circRNAs: Circular RNAs
COG: Clusters of orthologous groups of proteins
DECs: Differentially expressed circRNAs
DEGs: Differentially expressed genes
DEMs: Differentially expressed miRNAs
FPKMs: The fragments per kilobase of transcript per million mapped reads
GC: Guanine and cytosine
gDNA: Genomic DNA
GO: Gene ontology
KEGG: Kyoto encyclopedia of genes and genomes
KOG: Eukaryotes clusters of orthologous groups of proteins
LBD: Lateral organ boundaries domain
MADS-box: MCM1, AGAMOUS, DEFICIENS and serum response factor
MRE: Mirna response elements
MUT: Mutant type
NC: Negative control
NLP: Nin-like protein
NPFs: Nitrate and di/tripeptide transporters
PCR: Polymerase chain reaction
qPCR: Quantitative real-time PCR
SD: Standard deviation
TFs: Transcription factors
WT: Wild-type
