# Supplementary figures and images for: Identification and characterization of CircRNA-associated CeRNA networks in moso bamboo under nitrogen stress
Source: BMC Plant Biol. 2023 Mar 14;23:142. doi: 10.1186/s12870-023-04155-5 (PMC10012455; doi:10.1186/s12870-023-04155-5)

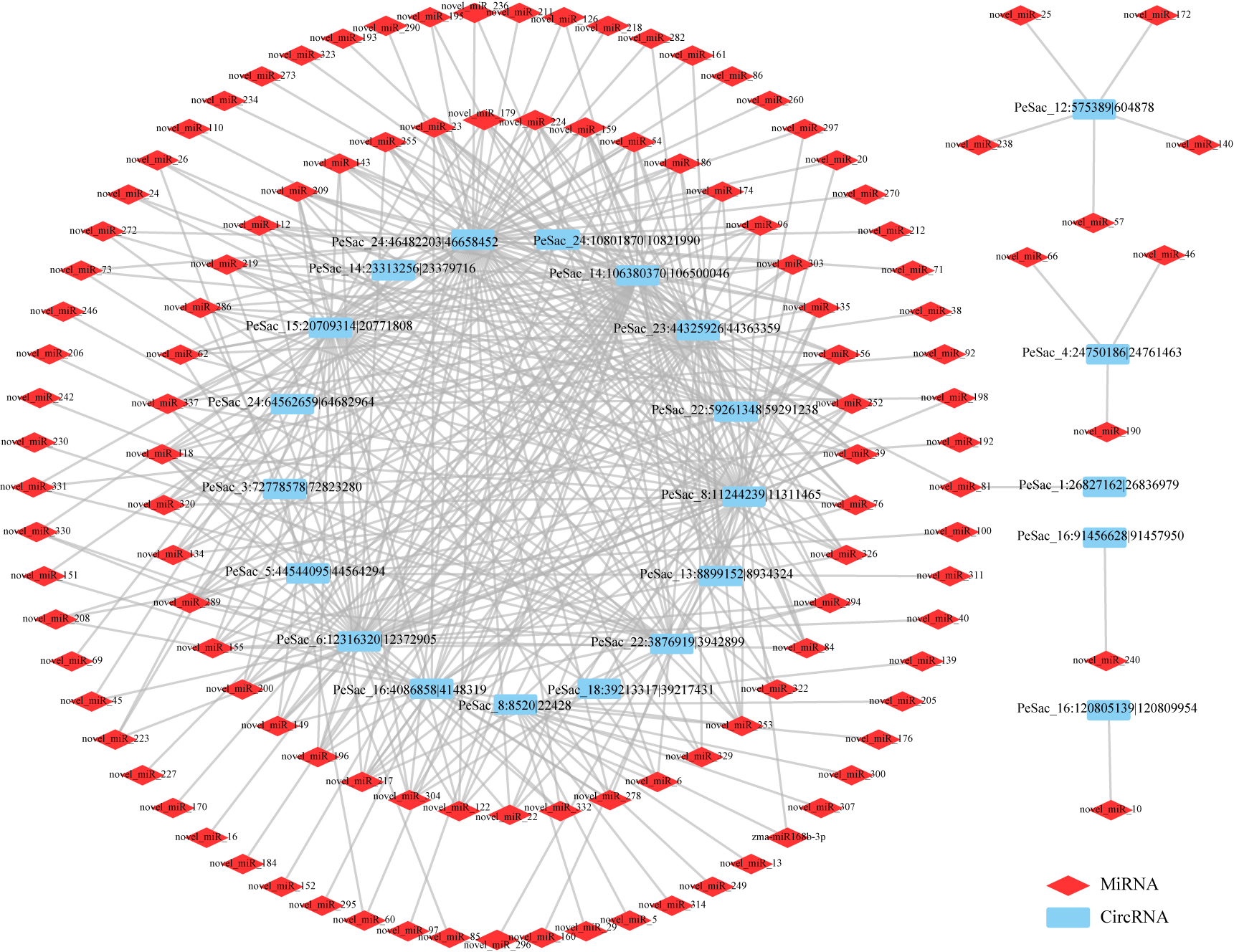

Supplement: Supplementary file 6 — Additional file 6: Figure S1. The circRNA-miRNA networks comprising the 22 DECs (blue square) and their targets of 118 miRNAs (red rhombus) in moso bamboo. [file 12870_2023_4155_MOESM6_ESM.tif]
